# Antioxidant Activities of Natural Polysaccharides and Their Derivatives for Biomedical and Medicinal Applications

**DOI:** 10.3390/antiox11122491

**Published:** 2022-12-19

**Authors:** Lu Bai, Dong Xu, Yan-Ming Zhou, Yong-Bo Zhang, Han Zhang, Yi-Bing Chen, Yuan-Lu Cui

**Affiliations:** 1 State Key Laboratory of Component-Based Chinese Medicine, Research Center of Traditional Chinese Medicine, Tianjin University of Traditional Chinese Medicine, Tianjin 301617, China; 2 Haihe Laboratory of Modern Chinese Medicine, Tianjin 301617, China; 3 Medical Experiment Center, First Teaching Hospital of Tianjin University of Traditional Chinese Medicine, Tianjin 300381, China; 4 Tianjin Key Laboratory of Translational Research of TCM Prescription and Syndrome, Tianjin 300381, China; 5 National Clinical Research Center for Chinese Medicine Acupuncture and Moxibustion, Tianjin 300381, China

**Keywords:** natural polysaccharides, antioxidant, reactive oxygen species (ROS), drug delivery, scientometrics

## Abstract

Many chronic diseases such as Alzheimer’s disease, diabetes, and cardiovascular diseases are closely related to in vivo oxidative stress caused by excessive reactive oxygen species (ROS). Natural polysaccharides, as a kind of biomacromolecule with good biocompatibility, have been widely used in biomedical and medicinal applications due to their superior antioxidant properties. In this review, scientometric analysis of the highly cited papers in the Web of Science (WOS) database finds that antioxidant activity is the most widely studied and popular among pharmacological effects of natural polysaccharides. The antioxidant mechanisms of natural polysaccharides mainly contain the regulation of signal transduction pathways, the activation of enzymes, and the scavenging of free radicals. We continuously discuss the antioxidant activities of natural polysaccharides and their derivatives. At the same time, we summarize their applications in the field of pharmaceutics/drug delivery, tissue engineering, and antimicrobial food additives/packaging materials. Overall, this review provides up-to-date information for the further development and application of natural polysaccharides with antioxidant activities.

## 1. Introduction

With the increasing pressure of life and the pollution of the surrounding environment, the incidence of chronic diseases is also increasing and showing a younger trend, including Alzheimer’s disease [1], diabetes [2], and cardiovascular diseases [3]. The study shows that these diseases’ occurrence is related to ROS imbalance [4,5]. Normal ROS can maintain the oxidative balance in the body, while excessive ROS breaks the dynamic balance of ROS, creating oxidative stress. ROS can attack cell membranes, affect the expression of nucleic acids, and eventually lead to apoptosis [6,7]. Natural products alleviate the excessive ROS caused by environmental, toxicological, and other factors by regulating enzyme activity and signal transduction pathways to exert antioxidant activity [8]. Compared to organic antioxidants, natural products present better biosafety during treatment [9,10].

Natural polysaccharides are a type of natural biomacromolecule found in plants, fungi, algae, animals, and bacteria [11]. Due to their nontoxic, stable, biodegradable, biocompatibility, and excellent antioxidant activity, natural polysaccharides contribute to the potential value in treating or preventing disease caused by oxidative stress [12]. Polysaccharides can reduce the damage to the cell structure, regulate the signal pathways related to antioxidation, improve the intracellular antioxidant enzyme system, reduce the substances that easily produce ROS, and protect the body tissue from ROS-induced damage through free radical scavenging activity and immunomodulatory activity [13,14]. Natural polysaccharides play an irreplaceable therapeutic role and have received more and more attention in recent decades. Publications related to natural polysaccharides are also increasing year by year [15,16,17]. Therefore, it is of great significance for future research to review and analyze relevant studies.

Scientometrics is a technical method commonly used to study the development status and hot trends of a certain field [18]. Several software tools have been developed for scientific literature research, such as Citespase and VOSviewer [19,20]. Scientometrics is also an effective technique to track the development trend of the field quickly. For example, Xu et al. used scientometric analysis to analyze the field of depression and found that research on the hippocampus will be its research hotspot in the future [21]. Therefore, using scientometrics to discover the research hotspot of natural polysaccharides will be a good method to discover the research hotspot of natural polysaccharides.

In this review, we confirm that antioxidant activity is a hot topic among natural polysaccharides by scientometric analysis. The antioxidant activity of natural polysaccharide and their derivatives will be discussed, and their applications in the field of biology and medicine will be summarized. These results will provide direction and reference for the antioxidant research of natural polysaccharides.

## 2. Scientometric Analysis of Polysaccharides and Their Antioxidant Activities

### 2.1. Search and Filtering of Bibliometric Data

Polysaccharide * and polysaccharide * and antioxidant as two topic words are searched in China National Knowledge Infrastructure (CNKI), PubMed, and Web of Science (WOS) from 1 January 2001 to 31 December 2021

### 2.2. Download of Bibliometric Data

The number of annual publications is collected in CNKI, PubMed, and WOS. The highly cited articles and reviews in the WOS are refined for bibliometric analysis. The record contents of the original data are full records and cited references in plain text file format.

### 2.3. Methods of Bibliometric Analysis

The bibliometric data are analyzed using bibliometrix (Version 3.1.4) in the R project (Version 4.1.2) and VOSviewer (Version 1.6.16), respectively. All keyword information is extracted by bibliometrix package.

### 2.4. Results and Discussion

#### 2.4.1. Bibliometrics of Papers Based on CNKI, PubMed, and WOS Databases

**Figure 1:**
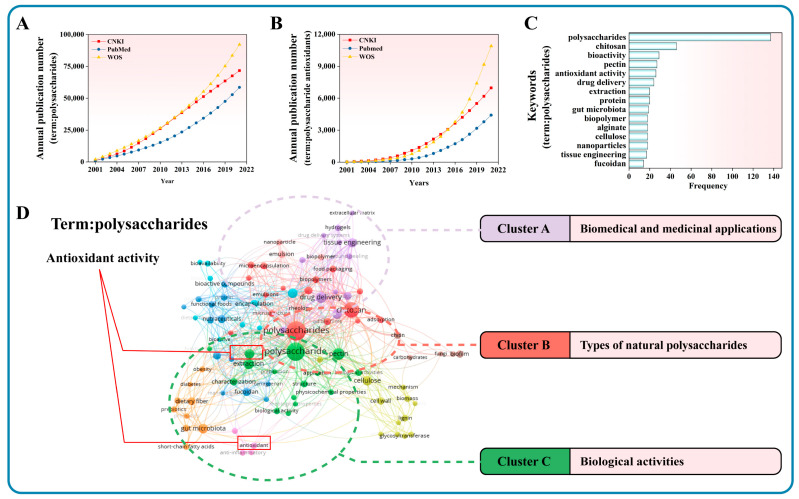
Scientometric analysis of polysaccharides. (**A**) Search results with polysaccharides as the topic word; (**B**) Search results with polysaccharides and antioxidant. (**C**) Frequency of keywords. (**D**) Keywords co-occurrence network. Node size and color represent the number of keywords and clusters. Lines of different colors represent different clusters.

According to the search results of CNKI, PubMed, and WOS databases, the studies of polysaccharides have increased in a large gradient since 2010 (Figure 1A). Since 2010, articles on polysaccharide antioxidants have increased and a substantial increase in this yield has appeared from 2015 (Figure 1B). The search results of polysaccharides and their antioxidants as topics show a similar increasing trend, which indicates that the research onpolysaccharides has increasingly become a hotspot. Antioxidant activities, as a hotspot research direction for polysaccharides, have gradually attracted the attention of some researchers.

#### 2.4.2. Keywords Analysis of Highly Cited Papers

According to the theory of bibliometrics, keywords represent the hot spots and trends in a research field [22]. Highly cited papers are considered as representative of research hotspots in a field and have literary influence. Therefore, keywords analysis of highly cited papers more accurately captures research hotspots. Polysaccharides are used as the topic to search for highly cited papers in WOS. The top fifteen keywords with the highest frequency in highly cited papers are counted (Figure 1C). The hot keywords mainly include polysaccharides, chitosan, bioactivity, pectin, antioxidant activity, drug delivery, etc. For the application of polysaccharides, the drug delivery system is more attractive. As the only research object in the direction of biological activity, Antioxidant activity plays a leading role in the research of natural polysaccharides. In addition, we use the VOSviewer software to create the high-frequency keywords co-occurrence network clustering map (Figure 1D). It can be seen that the largest node is polysaccharides, which is the same as the research topic. Keywords’ co-occurrence networks are divided into several clusters. Cluster A includes biomedical and medicinal applications, nanoparticles, tissue engineering, food packaging, etc. Cluster B contains types of polysaccharides, namely chitosan, chitin, etc. Cluster C has biological activities of polysaccharides, antioxidant, anti-inflammatory, etc. Through the classification of keyword hotspots, research hotspots are obtained in natural polysaccharides. The hotspot direction mainly includes the development of biomedical and medicinal applications, such as drug delivery, and the biological activities of some polysaccharides, such as antioxidant activity.

## 3. Antioxidant Mechanism of Natural Polysaccharides and Their Derivatives

Most ROS are radicals and others are molecules, such as hydrogen peroxide (H_2_O_2_) and singlet oxygen. A series of signal cascades maintains the redox homeostasis of cells in organisms [23]. Free radicals have high oxidative activity and are extremely unstable. They can attack cell and mitochondrial membranes, react with unsaturated fatty acids in the membranes, and enhance lipid peroxidation. Lipid peroxide can decompose into more free radicals and cause a chain reaction of free radicals. Oxidative stress induced by excess ROS can cause damage to the body, resulting in various diseases, such as tumors, cardiovascular disease, neurological diseases, hyperglycemia, etc. Natural polysaccharides and their derivatives with strong antioxidant capacity can remove excess free radicals [24,25].

The chemical structure is closely related to biological activity. Natural polysaccharides usually exist together with other components, such as proteins and polyphenols. More and more studies show that this may be part of the reason why natural polysaccharides have antioxidant effects. For example, Pu’erh tea polysaccharides with the highest total phenol and protein content were reported to have the best antioxidation activity [26]. In another study on *Gastrodia elata* polysaccharides, it was shown that polysaccharides containing higher binding proteins and polyphenols (GaE-R) had higher antioxidant activity than polysaccharides containing higher total sugars (GaE-G) [27]. In addition to proteins and polyphenols, uronic acid in *Litchi* polysaccharides and *Ampelopsis grossedentata* polysaccharides have also been proven beneficial to antioxidant activity [28,29]. Siu et al. found that the purified polysaccharide component with phenol and protein removed has no significant antioxidant activity in the study of edible *mushroom* polysaccharides [30]. These results indicate that the phenols and protein structure contained in crude polysaccharides may be the important reason for their antioxidant activity. In the study of *Tremella fuciformis* polysaccharides, both polysaccharides containing protein and uronic acid have different antioxidant activities. That may be due to their different average molecular weight and monosaccharide ratio [31]. Therefore, not all polysaccharides binding proteins and polyphenols have higher antioxidant activity, which is also affected by the average molecular weight of polysaccharides and the composition and proportion of monosaccharides. In addition, natural polysaccharides also exert antioxidant effects by regulating the signal transduction pathway, the activity of enzymes, and scavenging free radicals or substances that easily form free radicals.

### 3.1. Natural Polysaccharides Exert Antioxidant Effects by Regulating Signal Transduction Pathways

The regulation of the nuclear factor-E2-related factor 2 (Nrf2) signal pathway is an important way to alleviate ROS-induced injury. Under normal conditions, Nrf2 binds to Kelch-like ECH-associated protein-1(Keap1) and degrades rapidly, then maintains a low level in the cytoplasm. Nrf2 is released from Keap1 when antioxidant polysaccharides are supplemented. Then, Nrf2 enters the nucleus, interacts with the antioxidant response element (ARE), and promotes the expression of downstream antioxidant protein and a phase II detoxification enzyme [32]. It was reported that *Ostrea rivularis* polysaccharide could increase the expression level of Nrf2 and its downstream ARE gene in the testis, and enhance the Keap1-Nrf2/ARE signal pathway to avoid male reproductive dysfunction [33]. It was found that sulfated *Cyclocarya paliurus* polysaccharides (CPP) significantly increased Nrf2 protein, decreased Keap1 protein, and achieved an antioxidant effect by regulating the keap1/Nrf2 pathway. In addition, compared with bare CPP, sulfated CPP has better antioxidant activity in reducing oxidative damage [34]. Alternatively, the Nrf2 signaling pathway is activated by regulating its upstream molecules, such as glycogen synthase kinase 3β (GSK-3β) [35]. In addition to promoting Nrf2 translocation, natural polysaccharides also upregulate the expression of PI3K and AKT phosphorylation, inhibit the expression of JNK and IRS1, and effectively improve the degree of oxidative damage [36,37].

Oxidative stress can be induced by H_2_O_2_, which leads to the release of cytochrome C from mitochondria [38]. Mitochondrial damage disrupts the energy metabolism of chondrocytes and then induces chondrocyte apoptosis. *Angelica sinensis* polysaccharides can increase the expression of Bcl-2 and Bcl-xL by inhibiting the caspase pathway to play an antioxidant role and protect chondrocytes from oxidative stress-induced damage [39]. *Schisandra chinensis* polysaccharides (SCP) regulate protein expression in the mitogen-activated protein kinase (MAPK) pathway and the mitochondria-dependent apoptosis signaling pathway to exert an antioxidant effect. Antioxidant polysaccharides can downregulate the proteins of p-jnk1, p-ERK1/2, p-p38, Bax, caspase 3, and cytochrome C and upregulate the mechanism of Bcl-2 protein [40]. In addition, the curative effect of selenium-modified SCP is better than SCP.

### 3.2. Natural Polysaccharides Exert Antioxidant Effects by Regulating the Activity of Enzymes

Natural polysaccharides can exert antioxidant effects by indirectly acting on antioxidant enzymes. Endogenous antioxidant enzymes form the first line of defense against antioxidants in the body, such as superoxide dismutase (SOD), catalase (CAT), and glutathione peroxidase (GSH-Px) [41]. SOD specifically removes superoxide anion free radicals and converts superoxide anion free radicals into H_2_O_2_ through a reduction reaction. CAT is a terminal oxidase that catalyzes the decomposition of H_2_O_2_ in cells. GSH-Px can scavenge H_2_O_2_ and lipid peroxide. CAT and GSH-Px decompose H_2_O_2_ into non-toxic water molecules, and GSH-Px catalyzes the conversion of GSSG into non-toxic GSH. Natural polysaccharides and their derivatives enable regulation of the activity of the above antioxidant enzymes by affecting signaling pathways such as Keap1/Nrf2/ARE to play the role of antioxidant [42,43].

In addition to activating antioxidant enzymes, polysaccharides also restrain oxidases such as inducible nitric oxide synthase (iNOS). Overexpression of iNOS can produce many hydroxyl radicals and NO_2_ radicals and stimulate oxidative stress. Polysaccharides can significantly improve antioxidant capacity by inhibiting the expression of iNOS. For example, pretreatment with *Angelica sinensis* polysaccharides (ASP) before treating human osteoarthritis (OA) chondrocytes with H_2_O_2_ can reduce the activity of iNOS and alleviate the degree of oxidative stress injury [44].

### 3.3. Natural Polysaccharides Exert Antioxidant Effects by Scavenging Free Radicals or Substances That Easily Form Free Radicals

The hydroxyl and side-chain glycosidic bonds in the polysaccharides act as electron donors to bind free radicals and free radical ions. That is to say, hydrogen atoms on the polysaccharide chain can combine with various ROS to form water or be scavenged by oxidation reactions.

Polysaccharides or polysaccharide derivatives whose structures contain the following two or more functional groups: −OH, −O−, −COOH, −NR_2_, C=O, and −S− have exemplary structure–function configuration and show metal chelating activity [45]. Polysaccharides reduce the redox potential, stabilize the oxidation form of metal ions, prevent the production of excessive free radicals, and protect the body from oxidative stress by chelating metal ions [25].

**Figure 2:**
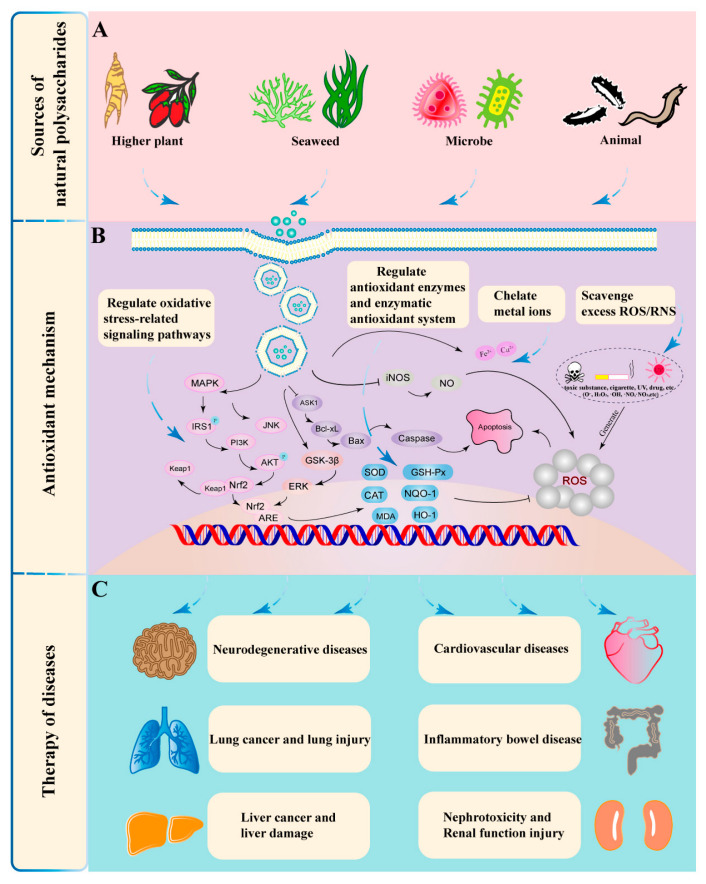
An overview of natural antioxidant polysaccharides. (**A**) The four types of polysaccharide sources; (**B**) the antioxidant mechanism of polysaccharides and their derivatives; (**C**) the therapeutic effects of natural polysaccharides on ROS-induced diseases.

Molecular weight, configuration, and solubility affect the antioxidant effect of natural polysaccharides, which can be regulated by molecular modification, especially chemical modification. Sulfated polysaccharides are formed by replacing the hydroxyl, carboxyl, or amino ends of polysaccharides with sulfate groups. Sulfate groups can enhance the ability of polysaccharides to provide hydrogen atoms, which is conducive to improving the antioxidant activity of polysaccharides [46]. Similarly, phosphorylation-modified polysaccharides can be obtained by replacing the hydroxyl groups on the polysaccharide chain with phosphate groups. The obtained polysaccharides have enhanced antioxidant activity. Many natural polysaccharides with weak biological activity improve their antioxidant properties by increasing water solubility and changing molecular weight [47]. The acetylation reaction is completed by acetyl replacing the hydrogen atom of the hydroxyl in the polysaccharide ring. The branches of the polysaccharide chain are prolonged, and the polysaccharide structure is changed. Therefore, the water solubility of the polysaccharide is improved, then its antioxidant activity is improved [48]. An overview of natural antioxidant polysaccharides is shown in Figure 2.

## 4. Antioxidant Activity of Natural Polysaccharides

### 4.1. Higher Plant Polysaccharides

Higher plant polysaccharides have the highest proportion of natural polysaccharides. They are also the type of natural polysaccharide with the most abundant sources, convenient extraction, and extensive research. It has been reported that plant polysaccharides have many pharmacological effects, such as anti-tumor and anti-diabetes effects. These pharmacological effects are closely related to antioxidant function.

*Lycium barbarum* polysaccharides (LBP) are the most bioactive polymer in *Lycium barbarum* fruit extracts and are widely used for food and medical treatment. Due to its multiple pharmacological effects, such as antioxidant, anti-cancer, anti-diabetes, and neuroprotection, LBP has been extensively studied in various disease models. The content of LBP is an important factor affecting the efficacy of *Lycium barbarum* [49]. Studies have shown that LBP can play an antioxidant role by removing superoxide anions from mitochondria in cells through an oxidation reaction. In addition, four molecular weight polysaccharide components were extracted from LBP using an integrated tandem hybrid membrane technology, and all of them have antioxidant activity. Ferric-reducing antioxidant power (FRAP) assay showed that the lower molecular weight polysaccharide component (LBP4 MW = 38 kDa) has more significant antioxidant activity. Moreover, the increase in polysaccharide concentration is also helpful to scavenge superoxide free radicals. As such, the four polysaccharides have scavenging activity that is maximal at 2.0 mg/mL [50]. LBP also indirectly acts on free radicals by capturing ROS produced during the peroxidative chain reaction. The polysaccharides capture ROS to reduce the level of lipid peroxidation and alleviate oxidative damage [51].

Some researchers have made the first study on the antioxidant activity of *Astragalus* polysaccharides (AP). The results showed that APs contain mannose, glucose, arabinose, and galactose, and their proportions decreased in order. Compared with normal rats, AP-treated rats could significantly inhibit the production of malondialdehyde (MDA) and increase SOD, CAT, and GSH-Px levels in the blood and liver [52]. Many factors affect the antioxidant activity of polysaccharides, such as the extraction method. Some researchers have evaluated the effect of extraction methods on the antioxidant activity of APs. By comparing hot water extraction, ultrasonic-assisted extraction, enzyme-assisted extraction, and enzyme ultrasonic-assisted extraction, some researchers found that the polysaccharides extracted by enzyme-assisted extraction had the highest extraction rate and antioxidant activity [53]. Recently, it was found that APs can reduce the side effects of tilmicosin in rats, mainly because APs can relieve tilmicosin-induced oxidative stress by causing a significant decline in the levels of MDA and other oxidative stress indexes [54].

*Dendrobium officinale* is a type of precious traditional Chinese medicine that has a remarkable curative effect on treating pharynx and lung diseases. In terms of pharmacological effects, flavonoids are found to have vital antioxidant activities [55]. In addition to flavonoids, *Dendrobium officinale* polysaccharides (DOP) also have antioxidant activity. Some studies found DOPs with a smaller molecular weight have more potent antioxidant activity through antioxidant tests such as DPPH radical scavenging, ABTS radical scavenging, and hydroxyl radical scavenging [56]. In addition to the influence of molecular weight, DOP obtained by the freeze-drying method (FD) has the best antioxidant effect [57]. Some researchers purified the crude DOP by chromatographic separation. It was concluded that the main monosaccharide components in the purified polysaccharides were mannose and glucose, which protect RAW 264.7 macrophages by resisting H_2_O_2_-induced oxidative damage [58].

As a well-known blood-tonifying medicine, *Angelica sinensis* has good pharmacological effects, such as anti-oxidation and anti-tumor effects. ASP is one of the vital active ingredients for these pharmacological effects. Recently, studies have explored the antioxidant effect of ASPs on human OA chondrocytes induced by H_2_O_2_. It was found that compared with normal human chondrocytes, OA chondrocytes produce more ROS, NO, and MDA, while ASP increases the activity of iNOS, SOD, and CAT [44]. Studies have also shown that ASP can protect H9c2 cells from apoptosis induced by H_2_O_2_. The results are because ASP inhibits the production of ROS and lactic dehydrogenase release by activating the ATF6 pathway, thereby improving oxidative stress and endoplasmic reticulum stress levels [59]. Tian S et al. studied the antioxidant activity of ASPs obtained by the best extraction process. Taking ascorbic acid as the positive control group, they studied the effect of ASPs at different concentrations on the scavenging of several common free radicals. The results showed that the scavenging ability of hydroxyl free radicals was closely related to the concentration. When the maximum measured concentration was 800 mg/mL, the clearance rate was about 60%, close to ascorbic acid [60].

*Gastrodia elata* has many active components, such as gastrodin and luteolin. Since Liu reported another active component, *Gastrodia elata* polysaccharides (GP), in 1981 [61], more and more studies on GP have been reported, such as the modification of the structure [62], the neuroprotective effect [63], regulation of intestinal flora [64], and immune enhancement [65]. These research results showed that GP has excellent pharmacological effects. Recently, the anti-aging activity of GP has gradually attracted people’s attention. One study extracted GP by the hot water extraction method, and chemical characterization by UV spectrometer and HPLC, then detected its antioxidant activity in vitro and in vivo. The results showed that GP could effectively remove hydroxyl radicals and has a substantial reduction ability [66]. Oral GP can increase the activities of SOD and GSH-Px in aging mice induced by D-galactose. Based on the above results, it can be concluded GP has high antioxidant activity and is beneficial in alleviating human aging.

*Purslane* polysaccharides (PP) can promote the production of serum cytokines and enhance the immune response in gastric cancer rats induced by N-methyl-N’-nitro-N-nitrosoguanidine (MNNG). PP plays an antioxidant role by increasing the activities of SOD, CAT, and GSH-Px. In addition, the protective effect on oxidative damage is dose-dependent [67]. Other researchers specially studied the free radical scavenging characteristics of PP by spectrophotometry. It was found that PP composed of glucose and galactose could scavenge superoxide anion free radicals, hydroxyl free radicals, DPPH free radicals, and NOs [68]. Free radicals have strong oxidizability, so they have a strong electron absorption ability, which will snatch the electrons of adjacent molecules and cause oxidative damage. Therefore, the free radical scavenging ability of PP has a certain application value in the antioxidation effect of diseases.

*Polygonatum sibiricum* polysaccharides (PSP) are considered to have an indispensable role in the treatment of diseases such as fatigue and diabetes [69]. Recent literature reported that PSP could also affect depressive behavior [70]. It was found that taking PSP can reduce the decrease of hippocampal 5-HT levels and hippocampal oxidative stress induced by lipopolysaccharides. Comprehensive analysis shows that PSP may treat depression by inhibiting ROS/HPA axis hyperfunction and inflammatory response, which can be attributed to the antioxidant effect of PSP. Some researchers specially studied the antioxidant effect of PSP [71]. In vitro tests show that the components isolated from PSP had the highest hydroxyl radical scavenging rate (78.585%) at 5 mg/mL concentration and had a strong ability to scavenge ABTS and DPPH radicals with scavenging rates of 92.794% and 75.648%, respectively. In addition, the power of chelating Fe^2+^ was up to 98.721%, which is in sharp contrast with other components. *Polygonatum odoratum*, similar to *Polygonatum sibiricum*, is also a *Liliaceae* plant with an antioxidant capacity [72]. For example, studies have explored the effect of polysaccharides isolated from *Polygonatum odoratum* (PPO) on oxidative stress induced by fatigue exercise. They have detected the changes in various biochemical parameters in serum and liver. PPO stimulates a protective effect on oxidation induced by fatigue exercise by reducing lipid peroxidation, which is attributed to the antioxidant effect of PPO.

### 4.2. Algal Polysaccharides

Algae are a class of substances with excellent nutrients in the ocean and are a crucial edible and medicinal resource. Polysaccharides are the main component of algae. Many biological activities of algae are closely related to algal polysaccharides. The monosaccharide composition, proportion, and molecular weight of algal polysaccharides are closely related to their biological activities, especially the sulfuric acid groups.

Five kinds of algal polysaccharides, *Ulva pertusa*, *Laminaria japonica*, *Enteromorpha linza*, *Bryopsis plumose*, and *Porphyra haitanensis* have been extracted and tested by in vitro antioxidant activity [73]. The results showed that all samples had antioxidant activity and strong free radical scavenging ability, and their scavenging effect could remain stable at high temperatures. In the test results of reduction ability, *Laminaria japonica* polysaccharides (LJP) showed the most potent reduction ability, which may be because LJP contains more sulfate and less hydroxyl, so fewer hydrogen atoms can be provided. The time-saving and straightforward microwave-assisted extraction technology is a commonly used extraction method for algae polysaccharides. The composition, properties, and biological activities of polysaccharides prepared by different extraction conditions are different and the optimal extraction conditions can be determined by response surface methodology. Fucoidan extracted from brown algae is a type of sulfated polysaccharide. The best extraction method is to extract for 15–17 min at a temperature of 120 °C and then obtain fucoidan with a concentration of 16%. The DPPH scavenging and reducing power measurement results showed that the fucoidan extracted at 90 °C has the highest antioxidant activity [74]. Similarly, *Ulva pertusa* polysaccharides were also extracted by microwave-assisted extraction technology [75]. The results of DPPH and ABTS showed that they had a strong antioxidant capacity and could improve H_2_O_2_− induced oxidative stress by increasing the activities of SOD and CAT.

Compared with green algae and red algae, brown algae have better antioxidant activity. Some studies have extracted fucoidan, laminaran, and alginate polysaccharides from Malaysian brown seaweeds and compared their antioxidant activities [76]. Among them, *laminaran* polysaccharides from *Sargassum polycystum* have the highest superoxide anion scavenging ability, and alginate has high DPPH scavenging activity. It shows that *Sargassum polycystum* polysaccharides may be a promising natural antioxidant. Alginate was extracted and isolated from the *Sargassum polycystum*. Lipid peroxidation levels and antioxidant enzyme activities were measured in arthritis rats to analyze whether alginate alleviates oxidative stress. The treatment of alginate can reduce the activity of various enzymes, and its antioxidant effect helps reduce inflammation in arthritis rats [77]. In addition, before the extraction of fucoidan and alginate, different fungal fermentation treatments enhance their antioxidant capacity [78]. After treatment, the molecular weight of the two polysaccharides decreased by nearly half. The content of fucose and sulfate in fucoidan and the ratio of mannuronic acid to guluronic acid (M/G) in alginate increased, suggesting that molecular weight and monosaccharide components may be the key factors affecting antioxidant capacity. Radiation is often used to degrade sodium alginate. Some researchers analyzed the effects of the ratio of M/G and the molecular weight of mannuronic acid and guluronic acid on the antioxidation ability. The results showed that the lower the molecular weight, the higher the 50% inhibition concentration [79]. Under the same molecular weight, the fraction with higher M/G has more potent antioxidant activity. It further shows that the molecular weight, content, and proportion of monosaccharide components are essential factors affecting the antioxidant properties of polysaccharides. In addition to radiation degradation, heat treatment depolymerization also obtained low molecular weight sodium alginate. ABTS and superoxide radical scavenging tests obtained similar results [80]. This suggests that low molecular weight sodium alginate is a rich and potential source of natural antioxidants.

### 4.3. Microbial Polysaccharides

#### 4.3.1. Fungal Polysaccharides

Fungal polysaccharides are natural polymer compounds isolated from the fruit body, or mycelium, of fungi. They are mainly derived from medicinal mushrooms. Their excellent antioxidant effect and safe properties enable them to be widely used in medicine, food, cosmetics, and other industries.

*Mushroom* polysaccharides are a large class of fungal polysaccharides, mainly including *Lentinus edodes* polysaccharides (LEP), *Flammulina velutipes* polysaccharides (FVP), *Pleurotus ostreatus* polysaccharides (POP), etc. Studies have extracted LEP with hot water extraction [81]. The results of scavenging hydroxyl free radicals, superoxide free radicals, and Fe^2+^ chelating capacity have verified that LEP has a dose-dependent antioxidant activity. There are also many reports on the effects of different molecular weights on the antioxidant activity of purified LEP [82]. By detecting the contents of various peroxidases and MDA in the liver, it was concluded that LEP has a protective effect on oxidative stress induced by D-galactose, and the effect of medium molecular weight is the best. To accurately assess the antioxidant capacity of LEP, some studies have adopted two methods of protein removal [83]. The results found that the deproteinized polysaccharides showed reduced antioxidant activity. The reduced antioxidant activity may be due to the hydrophobic cavity and fissure formed by the covalent bond between polysaccharides and protein. In addition, the study also showed that the neutral protease protein removal method was better for retaining the antioxidant activity of polysaccharides. The results of the antioxidant evaluation of polysaccharides by different extraction methods are very different. Some studies have compared the differences in antioxidant activity caused by different extraction methods of FVP [84]. It was found that enzymatic extraction has a strong hydroxyl radical scavenging and metal chelating ability, while ultrasonic extraction has a good effect on DPPH scavenging. The hot water extraction method has stronger antioxidant activity in terms of reduction ability. In another study, microwave-assisted extraction was used [85]. The effect of FVP on scavenging a variety of free radicals in vitro was analyzed. The results showed that the acid polysaccharides rich in glucose and the low average molecular weight components had stronger antioxidant potential. A study compared the relationship between the structural characteristics and antioxidant activity of polysaccharides extracted from *Pleurotus eryngii*, *Flammulina velutipes*, *Pleurotus ostreatus*, and white *Hypsizygus marmoreus* [86]. It was found that POPs rich in acid groups and with low molecular weight had the best antioxidant effect. Similarly, another study on extracting and isolating four polysaccharides from *Agaricus bisporus*, *Auricularia auricula*, *Flammulina velutipes*, and *Lentinus edodes* also showed that the polysaccharides of *Agaricus bisporus* with a low molecular weight had the most potent antioxidant activity [87].

In addition to the common *mushroom* polysaccharides, *Grifola frondosa*, a rare medicinal and edible fungus, has also attracted extensive attention. Some studies have extracted the *Grifola frondosa* polysaccharide (GFP) and separated three main polysaccharide components through chromatographic purification [88]. The results of in vitro antioxidant experiments showed that it has noticeable scavenging effects on various free radicals. In addition, strong reducing power and Fe^2+^ chelating ability were also observed. Based on these studies, some researchers have explored the effect of GFP on improving memory and anti-aging [89]. The behavioral experimental results suggested that GFP can significantly improve the memory ability of elderly rats. The pathological analysis also showed that the changes in brain histology and ultrastructure after aging are weaker, and the total antioxidant capacity and the activities of various antioxidant enzymes are improved. At the same time, it also reduced the levels of NO and MDA. The above results showed that GFP had an antioxidant ability and could effectively improve memory.

*Ganoderma lucidum* is widely popular because it contains a variety of highly bioactive compounds [90]. *Ganoderma lucidum* polysaccharides (GLP) with antioxidant effects have been extensively studied. Additionally, similar to the optimum drying method of other polysaccharides, the vacuum freeze-drying method used on GLP has a strong reducing power and free radical scavenging rate [91]. In many studies on the antioxidant effect of GLP, extraction methods occupy many of them. The early studies detected the antioxidant activity of ultrasonic-extracted GLP [92]. It was found that the four components separated by ultrafiltration membrane were rich in glucose and galactose. Recent studies have found that the antioxidant activity of GLP extracted by hot water extraction is stronger than that extracted by ultrasound [93]. The polysaccharides obtained by the two extraction methods have the same kind of monosaccharide, but the proportion of glucose in the polysaccharide extracted by hot water is about twice that extracted by ultrasound. There are also studies on extracting GLP by superfine grinding technology [94]. Among the three crude polysaccharides, the proportion of glucose is very similar. GLP 80 with the highest proportion of galactose has the highest antioxidant activity. Based on the above research results, this may be the result of the joint influence of the content and proportion of glucose and galactose.

In addition to the more researched fungi reviewed above, there are also other polysaccharides from *Hericium erinaceus* [95], *Cordyceps sinensis*, and *Ganoderma atrum* [96,97] that have good antioxidant activities. Activating antioxidant enzyme activity prevents mitochondrial-dependent apoptosis and plays a regulatory and protective role in colonic immune disorder and hepatotoxicity.

#### 4.3.2. Bacterial Polysaccharides

In addition to fungal polysaccharides, bacterial polysaccharides are also a part of microbial polysaccharides. Because they are more suitable for producing extracellular polysaccharides than plants, animals, and seaweed, they have gradually attracted people’s attention [98]. The extracellular polysaccharides extracted from *Lactobacillus plantarum* have no cytotoxicity. The clearance of DPPH and lipid peroxidation can reach 64% and 66%, respectively [99]. The extracellular polysaccharides of *Brevibacterium otitidis* BTS 44 with good antioxidant activity were determined by in vitro antioxidant test [100]. The components were mannose, arabinose, glucose, and mannouronic acid, and the proportion decreased successively. An extracellular polysaccharide was extracted and purified from *Bacillus* sp. strain LBP32, which can alleviate LPS-induced inflammation by inhibiting oxidative stress [101]. *Streptomyces* isolated from soil can produce an extracellular polysaccharide mainly composed of mannose and glucose [102]. It can effectively scavenge hydroxyl radicals and DPPH radicals and has a high Fe^2+^ chelation ability. Various results showed that the extracellular polysaccharides produced by *Streptomyces* had good antioxidant activity. Some researchers studied the antioxidant activity of extracellular polysaccharides of *Streptomyces violaceus* MM72 from marine actinomycetes and obtained similar results [103]. The above results show that bacterial polysaccharides may be a good source of natural antioxidants.

### 4.4. Animal Polysaccharides

Animal polysaccharides are mainly derived from marine animals. Because of the unique living environment of marine animals, marine animal polysaccharides have unique structures and characteristics [104]. For example, the contents of sulfated polysaccharides in marine animal polysaccharides are higher than that in terrestrial animal polysaccharides. Many studies have proven that sulfated polysaccharides contribute to antioxidant activity [105]. In addition, compared with other marine organisms, marine animal polysaccharides usually exist in the form of covalent bonds and protein complexes. This particular structure also contributes to the antioxidant function [106].

*Misgurnus anguillicaudatus* polysaccharide (MAP) is a natural neutral polysaccharide in loach mucus. Early studies have proved that MAPs can scavenge various ROS, and their antioxidant activity can significantly reduce damage to the DNA chain by hydroxyl radicals. Based on this, some researchers have studied the therapeutic effects of MAP on diabetes and found that MAP can significantly scavenge superoxide anion free radicals and hydroxyl free radicals in a dose-dependent manner [107]. The results showed that its antioxidant and anti-glycosylation capabilities could protect the body from diabetes-induced oxidative damage.

Two kinds of chondroitin sulfate were extracted and isolated from two kinds of sea cucumber [108]. They can scavenge free radicals in a dose-dependent manner, which is suitable proof of the antioxidant capacity of animal polysaccharides.

Animal polysaccharides exist in mucus, cartilage, and the skin, such as *Rana chensinensis* skin. Some researchers have optimized the best process for extracting polysaccharides from *Rana chensinensis* skin (RCSP) [109]. Under the conditions of extraction temperature at 100 °C, water–material ratio of 60:1, extraction frequency of 1, and extraction time of 4.96 h, a yield of RCSP of about 2.03% can be obtained. In vitro experiments showed that RCSP had significant scavenging effects on superoxide anion and DPPH and showed a strong reducing ability. Similar results were obtained in another study [110]. The preliminary characterization experiment showed that RCSP was composed of glucose, galactose, and mannose (87.82:2.77:1.54), and its molecular weight was 12.8 kDa. The antioxidant experiment in vitro showed good free radical scavenging activity. The antioxidant experiment in vivo showed that it increased the antioxidant enzyme activity in the liver and serum of mice induced by D-galactose and decreased the level of MDA. Therefore, RCSP is a potent natural antioxidant that can be used in food and medicine.

**Figure 3:**
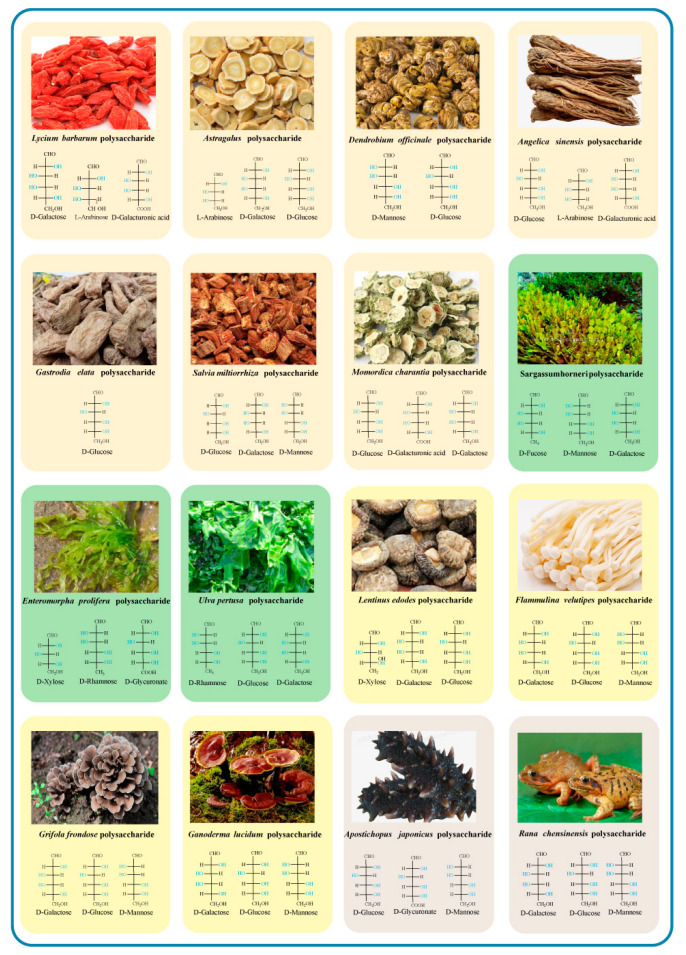
Sources and main monosaccharides of representative natural polysaccharides.

Chitosan is a natural polysaccharide with multiple biological activities. It can be produced from shellfish, shrimps, and crabs and can also be produced from microorganisms [111]. Chitosan is an excellent N-center free radical scavenger, which can scavenge peroxide free radicals and has a good antioxidant protection effect on human serum albumin [112]. Low molecular weight chitosan can inhibit neutrophil activation and play a significant role in serum albumin oxidation, reducing the oxidative stress associated with uremia. The sources and main monosaccharides of representative polysaccharides with antioxidant activity are shown in Figure 3 and the natural polysaccharides and their antioxidant effects widely studied in recent years are summarized in Table 1.

## 5. Antioxidant Activity of Natural Polysaccharide Derivatives

### 5.1. Sulfated Polysaccharides

Sulfated polysaccharides are important derivatives of polysaccharides. The most common methods are the chlorosulfonic acid-pyridine method (CSA-Pyr) [126], phenol-sulfuric acid method [127], sulfur trioxide pyridine method, and amino sulfonic acid-pyridine method (ASA-Pyr) [128,129]. The sulfation modification methods of neutral polysaccharides and acidic polysaccharides are different. Neutral polysaccharides can be dissolved in organic solvents and then sulfate esterification is used to obtain sulfated polysaccharides. Acidic polysaccharides have fewer hydroxyl groups and are difficult to dissolve in organic solvents, so they need to be reorganized with acidic resins and then sulfated to obtain sulfated polysaccharides with a high degree of substitution. The neutral polysaccharides and acidic polysaccharides of *Auricularia auricula* polysaccharides (AAP) were sulfated by sulfation [130]. The completion of sulfation was successfully proved by ion chromatography and infrared spectroscopy. The results of antioxidant experiments in vitro showed that both had stronger antioxidant activity than the natural AAP without sulfation modification, especially in scavenging hydroxyl free radicals and lipid peroxidation. Studies have been performed on the sulfation modification of *Flammulina velutipes* polysaccharides (FVP) [122]. The results of structural characterization and antioxidant experiments in vivo and in vitro showed that the characteristic functional groups and monosaccharide composition of FVP have changed. Studies also found sulfated FVP has more antioxidant effects than natural polysaccharides and could improve aging and inflammatory responses. Some researchers are also interested in the sulfation modification of plant polysaccharides [131]. Sulfated *Momordica charantia* polysaccharides with different substitution positions were synthesized by CSA-Pyr. It was found that C-6 substitution had more advantages than C-2 substitution through spectral analysis. In addition, both of them have more antioxidant activity than natural *Momordica charantia* polysaccharides. It is concluded that a high degree of substitution and medium molecular weight significantly affects improving antioxidant activity. The effects of sulfated polysaccharides on antioxidant activity from different angles are not all positive. For example, sulfated *pumpkin* polysaccharides with different degrees of substitution are only superior to *pumpkin* polysaccharides in scavenging superoxide anions. Their reducing power is lower than *pumpkin* polysaccharides, even with no scavenging ability to hydroxyl radicals [127]. There are studies on the sulfation modification of *Cyclocarya paliurus* polysaccharides. In a study, the derivatives with the best antioxidant properties have the lowest degree of substitution and the highest relative molecular mass and protein content [132]. The degree of substitution, relative molecular weight, sulfate content, and protein of modified polysaccharides combined may influence the degree of antioxidant. Among animal polysaccharides, the in vitro experiments of sulfated chitosan with different modified sites also showed more potent antioxidant activity than chitosan. The inhibition of deoxyribose oxidation was dose-dependent, which could effectively alleviate oxidative stress, but safety needs to be evaluated in vivo [133].

### 5.2. Phosphorylated Polysaccharides

Several commonly used phosphorylation reagents include phosphorus oxychloride, phosphate, phosphoric acid and anhydride, and phosphorus pentoxide. Generally, the characteristic peaks obtained by Fourier-transform infrared spectroscopy and nuclear magnetic resonance are used for qualitative analysis of polysaccharides [134]. Scientists have reported the phosphorylation modification of a variety of polysaccharides. For example, *pumpkin* polysaccharides were modified with phosphorus oxychloride-pyridine to obtain phosphorylated pumpkin polysaccharides [135]. Compared with unmodified *pumpkin* polysaccharides, phosphate-modified *pumpkin* polysaccharides have higher superoxide and hydroxyl radicals scavenging ability. Some studies came to similar conclusions about ginseng polysaccharides using the same method [136]. Studies on the phosphorylation of garlic polysaccharides indicated that the higher concentration of phosphorylated garlic polysaccharides, the stronger the antioxidant capacity within a certain range [137]. In the animal tissue oxidative damage protection experiment of phosphorylated *Momordica charantia* polysaccharides, the content of antioxidant enzymes in mouse serum, liver, and other tissues had increased [138]. These results indirectly proved that sulfated *Momordica charantia* polysaccharides had a strong antioxidant ability. This feature has also been proven in the *Pleurotus ostreatus* of fungal polysaccharides, which can effectively treat chemical liver toxicity by improving the antioxidant enzyme activity and reducing the content of MDA in the liver [139]. These provide strong evidence for the enhanced antioxidant activity of phosphorylated polysaccharides.

### 5.3. Carboxymethylated Polysaccharides

Carboxymethylation is a common modification method of polysaccharides to improve water solubility and biological activity. The aqueous medium and organic solvent methods are the most common synthesis methods of carboxymethylation. The aqueous medium method dissolves the polysaccharides with an alkaline solution, adds monochloroacetic acid (MCA) to mix and react for several hours, and adds an acid solution to adjust the pH to a neutral range. Ethanol is the precipitation solvent, and carboxymethyl polysaccharide derivatives can be obtained after dialysis and freeze-drying [140]. This method is unstable and takes a long time, but it is cheap and commonly used. The organic solvent method is to dissolve polysaccharides in organic solvents such as isopropanol or ethanol, and then add MCA for etherification reaction to obtain carboxymethyl polysaccharide derivatives [46]. The reaction is stable and rapid, but the price is relatively expensive with the toxic organic reagent. Carboxymethyl-modified *pumpkin* polysaccharides prepared by the aqueous medium method were proven to have a good scavenging ability of superoxide anion free radicals and hydroxyl free radicals (Liu & Huang, 2019). It has been reported that the carboxymethylated polysaccharides with a molecular weight of 354 kDa were obtained by the chemical modification of *Sargassum fusiforme* polysaccharides [141]. Compared with the unmodified *Sargassum fusiforme* polysaccharides, the modified carboxymethyl polysaccharides have a stronger free radical scavenging ability and total antioxidant activity. Similarly, the crude polysaccharides of *Enteromorpha prolifera* were degraded with hydrogen peroxide or ascorbic acid and modified by carboxymethyl [142]. The reaction conditions of carboxymethylation were optimized by the response surface method. The product was characterized and analyzed, and carboxymethylated *Enteromorpha prolifera* polysaccharides with a degree of substitution of 0.849 were obtained. The carboxymethylated *Enteromorpha prolifera* polysaccharides had stronger antioxidant activity evaluated by DPPH, hydroxyl radical, and superoxide anion radical scavenging ability. Similar conclusions have also been proven in the studies of various carboxymethylated polysaccharides [143].

### 5.4. Other Modification Methods

**Figure 4:**
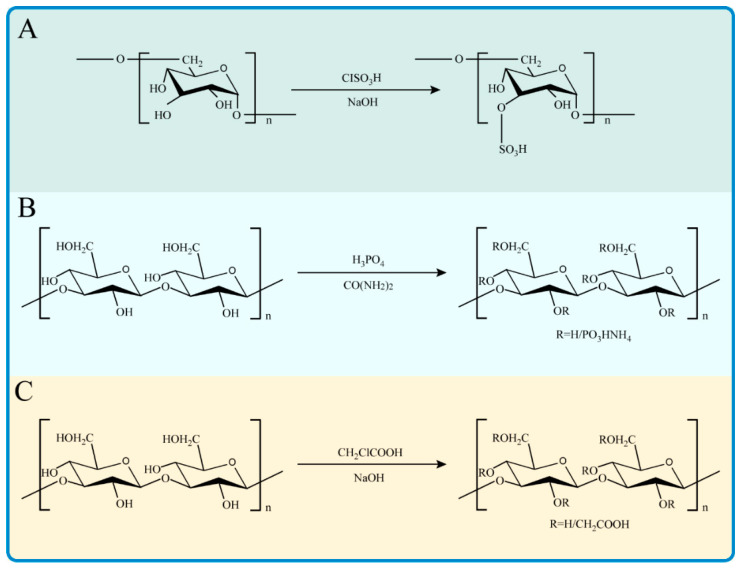
Several common modification methods and schematic diagrams of natural polysaccharides. (**A**) Sulfation modification with CSA; (**B**) Phosphorylation modification with phosphoric acid; (**C**) Carboxymethylation modification with monochloroacetic acid.

In addition, studies on multiple derivatives of the same polysaccharides also showed that the modified polysaccharide derivatives had better antioxidant activity than those without modification [144,145]. In addition to the above modification methods, acetylation modification, hydroxy-propylation modification, selenization modification, sulfonation modification, ammonium modification, and other modification methods can also affect the water solubility and biological activity of polysaccharides [138]. The commonly used chemical modification methods of natural polysaccharides are shown in Figure 4.

## 6. Biomedical and Medicinal Application of Natural Polysaccharides

### 6.1. Application of Natural Polysaccharides in Pharmaceutics/Drug Delivery

#### 6.1.1. Hydrogel

As a special material mainly based on the three-dimensional structure of cross-linked polymer networks, hydrogels are widely used in biomedicine and pharmaceutics. Due to the limitation of fossil sources of polymers, hydrogels based on natural polysaccharides and their derivatives are potential alternatives [146]. For example, various drug delivery systems based on natural gum polysaccharides and their derivatives were developed to improve the therapeutic effect of drugs. Some chronic diseases related to the gastrointestinal tract require long-term treatment. Some drugs for treating gastrointestinal diseases can also irritate the gastric mucosa and even cause systemic side effects. Natural gum polysaccharides have the characteristics of moisturizing and gelling and special properties such as antioxidants. Those properties make natural gum polysaccharides release drugs continuously and reduce damage to the body. In the presence of crosslinking agents, hydrogels modified by grafting hydrophilic monomers on polysaccharides were extensively studied in drug delivery systems. Some researchers have grafted polyvinyl pyrollidone (PVP) onto *Azadirachta indica* gum polysaccharides in the presence of a crosslinker and then prepared hydrogels. This design is to deliver methyl prednisolone to colon anti-inflammatory agents and achieve continuous targeted release [147]. The biocompatibility and antioxidant activity results showed that the polymer could scavenge free radicals and protect the oxidative damage of biological systems, which proved that the hydrogel carrier is suitable for colon administration.

Some studies on polysaccharide-based hydrogel drug delivery systems are more interested in the *Moringa oleifera* gum. The gum-based hydrogels were formed by radiation-induced graft-copolymerization of N-vinyl imidazole, poly (acrylic acid) (PVA), or acrylamide onto the gum [148,149,150]. In these studies, the pure and sterile gel-based hydrogels were characterized by cryo-scanning electron micrography and atomic force microscopy. The drug release curve showed that the *Moringa oleifera* gum polysaccharide-based hydrogel drug delivery system is suitable for delivering antibiotic drugs to the gastrointestinal tract, thus effectively treating gastrointestinal diseases. By measuring the properties of antioxidant and mucosal adhesion, it is found that the polymer is essentially antioxidant and mucus adhesive. Antioxidant materials can scavenge excess free radicals generated in the system and protect the system from oxidative damage, which is beneficial for biomedical applications. The antioxidant properties of these drug delivery systems are due to the presence of *Moringa oleifera* gum in the hydrogel matrix. The antioxidant activity of *Moringa oleifera* gum is mainly derived from the arabinogalactan in *Moringa oleifera* gum. This statement was further verified by applying arabinogalactan-based hydrogels to deliver the antibiotic drug ‘meropenem’ in gastrointestinal infections [151].

*Bletilla striata* polysaccharides are a natural glucomannan with antioxidant activity and biocompatibility, which is helpful for the biomedical application prospect of *Bletilla striata* polysaccharide-hydrogels. After carboxymethylation, *Bletilla striata* polysaccharides can form a multifunctional hydrogel with the dopamine conjugate catechol group, and its antioxidant properties and tissue adhesion are greatly improved. The encapsulated berberine exerts excellent and long-lasting antibacterial activity [152]. Oxidative stress in the brain leads to neuronal damage and necrosis, and antioxidants help reduce oxidative stress. Researchers developed a polysaccharide-based hydrogel film with biocompatibility, antioxidant properties, and mucoadhesive, so that the nerve-regenerating agent can be released from the drug-loaded polymer film and delivered to the brain [153]. It is of great significance to protect the cranial nerves.

#### 6.1.2. Nanoparticles

Many diseases or injuries are mostly related to oxidative damage, and the potent antioxidant activity of polysaccharide-modified nanoparticles enables them to have a wider potential application in medicine. Polysaccharides play the role of reducing agents and stabilizers in the preparation of nanoparticles so that they have antioxidant and antibacterial potential against certain wound pathogens or diseases [154].

Selenium nanoparticles (SeNPs) have attracted increasing attention due to their effects on redox balance in the human body. However, SeNPs tend to aggregate into large clusters, reducing bioactivity, bioavailability, and biocompatibility. Surface capping agents play a crucial role in the stability and biological activity of SeNPs, *Lycium barbarum* polysaccharides, and green tea extract not only shows the high scavenging ability of DPPH and ABTS but protects cells from oxidative damage induced by H_2_O_2_ [155]. Polysaccharides can be used as stabilizers and reducing agents of SeNPs. Compared with SeNPs without polysaccharides, polysaccharides-based SeNPs can exhibit higher antioxidant activity by scavenging free radicals [156]. Suppose the ratio of polysaccharides to SeNPs is appropriate. In that case, it can show a uniform and monodisperse spherical structure with a small average particle size and is more conducive to improving antioxidant activity [157]. The *Sargassum fusiforme* polysaccharides (SFPS) functionalized SeNPs (SFPS-SeNPs) were prepared by the chemical reduction method. SFPS-SeNPs have higher antioxidant activity, dispersion, and stability than naked SeNPs and SFPS with the same concentration [158]. As a modifier, SFPS significantly improves the free radical scavenging rate of SeNPs, because SFPS is an acidic polysaccharide rich in L-fucose. In addition, there are selenium esters in SFPS-SeNPs, which can stimulate the hydrogen supply capacity of non-monomeric regions, resulting in higher antioxidant activity. The problems of time-consuming, energy-consuming and high equipment requirements can be solved by ultrasonic extraction. Ultrasound can reduce the size of SeNPs and increase the specific surface area to provide enough active sites to react with free radicals and inhibit the oxidation reaction [159]. Polysaccharides with strong antioxidant activity can also be used as good reducing agents and stabilizers in Au-NPs [160] and Ag-NPs [161]. They are widely used in the treatment of skin cancer [162] and diabetes [163,164].

#### 6.1.3. Nanogel

Nanogels are also a reliable choice for drug delivery systems. Sodium alginate has been studied for various drug delivery systems. Some researchers constructed a self-assembled thermosensitive hydrogel system using alginate nanogels loaded with antidepressant albiflorin (Albiflorin-NGSTH) [165]. The gel presents gelation at about 28 °C. Albiflorin-NGSTH was administered intranasally to chronic unpredictable mild stress rats, and the results showed that the nanogel could improve the depressive behavior of depression model rats. DPPH test showed that an alginate gel loaded with albiflorin increased the antioxidant activity and showed higher antidepressant activity. Another study on the alginate gel delivery system improved the bioavailability of icariin [166]. Compared with oral administration, icariin can repair hippocampal neuron injury more effectively and play a rapid antidepressant role through the nasal–brain pathway.

Sodium alginate nanogels have also been used in liver disease. A report of acute liver failure mentioned that a glycyrrhizic acid-mediated liver targeting nanogel could effectively deliver quercetin to the liver. The antioxidant effect can be as high as 81 times that of naked quercetin [167]. Histopathological analysis showed that the nanogel had reversed liver injury. In another report on liver cancer, glycyrrhizin was introduced into sodium alginate nanogel and doxorubicin was loaded into this nanogel [168]. The nano gel shows good bioavailability and biocompatibility. By inhibiting the activation of macrophages and regulating the apoptosis pathway, the nanogel improves the therapeutic effect of liver cancer. Overall, these studies on nanogel drug delivery systems based on alginate provide an effective strategy for treating depression and other diseases.

#### 6.1.4. Protein Conjugates

**Figure 5:**
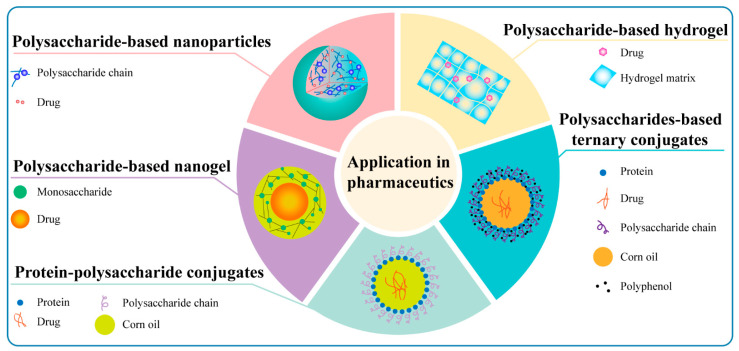
The application of natural polysaccharides in pharmaceutics or drug delivery.

Proteins are natural polyelectrolytes with the ability to produce emulsions. Natural polysaccharides can enhance the functionality and stability of proteins through their properties, such as antioxidants. Proteins combine with natural polysaccharides to form complexes for drug delivery and encapsulate drugs. The antioxidant capacity of the nanoparticles formed by encapsulating curcumin with soy protein isolate (SPI)-dextran conjugate can reach more than twice that of naked curcumin [169]. The enhanced antioxidant activity is not only because the structure of the compound is conducive to dispersing curcumin with low solubility in the reaction medium and reacting with free radicals in a larger area, but also because the polysaccharide itself has a certain antioxidant activity. SPI-*Pleurotus eryngii* polysaccharide conjugates improved in vitro digestion and cellular antioxidant activity of β-carotene-loaded emulsions and prepared emulsions with better physical and chemical stability [170]. Furthermore, polyphenols, polysaccharides, and proteins can be combined into ternary conjugates [171,172]. As antioxidants, the ternary conjugates can effectively capture free radicals, inactivate pro-oxidant transition metals as metal ion chelators, and physically inhibit the interaction between pro-oxidants and lipids. This characteristic can avoid the problem of droplet aggregation, emulsification, and phase separation in emulsions. The application of natural polysaccharides in pharmaceutics or drug delivery is shown in Figure 5.

### 6.2. Application of Natural Polysaccharides in Tissue Engineering

The structure and biological characteristics of polysaccharides make them suitable for becoming tissue engineering biomaterials. The anti-aging effect of some bacterial polysaccharides on the skin is helpful. This benefit is because the polysaccharides have antioxidant pharmacological activity against produced oxidative stress when dealing with ultraviolet radiation. As a natural compound of anti-elastase and anti-collagenase, *Lactobacilli* exopolysaccharides (LEP) could delay skin aging and maintain a healthy state by promoting fibroblast proliferation and scratch healing [173]. These studies provide evidence for LEP in tissue engineering and skin regeneration scaffold applications. 

Wound healing remains a medical challenge for patients with severe tissue damage from burns, diabetes, and ischemia [174]. If the host antioxidant capacity is insufficient to control the high levels of free radicals, cell migration and proliferation are inhibited, damaging the wound’s tissue. Therefore, applying natural polysaccharides and their derivatives with strong antioxidant activity would promote wound repair and healing [175]. As a hydrogel wound dressing, an exopolysaccharide hydrogel from *Pseudomonas stutzeri* AS22 (EPS22) was evaluated for its antioxidant activity by three different antioxidant activity tests [176]. The results of the DPPH scavenging ability, reducing ability, and Fe^2+^ chelating ability test proved that it has intense antioxidant activity. The antioxidant properties of EPS22 inhibited fibroblast death and decreased skin lipid elasticity and oxidative damage by protease inhibitors, which are one of the crucial factors in wound healing. In addition, after EPS22 hyperacetylation, the antioxidant activity is higher. *Ulvan* is a sulfated polysaccharide extracted from seaweed. It may become a candidate material for wound dressing because of its antioxidant, antibacterial, and other pharmacological activities. *Ulvan* polysaccharide chelates Fe^2+^ in a dose-dependent manner. The higher the concentration, the stronger the inhibitory effect on free radicals and the higher the antioxidant activity. It can not only absorb the wound fluid and prevent the accumulation of exudates, but also maintains a humid environment [177]. The composite hydrogel was prepared by crosslinking *Enteromorpha prolifera* polysaccharides (PEP) with boric acid and polyacrylamide [178]. The sulfate groups on PEP allowed for natural antioxidant properties. With the increase of PEP content in the hydrogel, the DPPH scavenging efficiency increased two-fold. This antioxidant activity is beneficial to the healing of skin wounds.

Activating growth factors and regulating the redox microenvironment are effective strategies to accelerate wound healing and regeneration in diabetic-impaired skin tissue. For example, 2-N,6-O-sulfated chitosan (26SCS), which has proven to have an antioxidant effect, can protect the human keratinocyte cell line (Ha-cat) from oxidative damage induced by H_2_O_2_ during reproduction [179]. Histopathological evaluation showed that treatment with a scaffold containing 26SCS could accelerate re-epithelialization and positively affect wound healing. Similar results were found in the study of *Flammulina velutipes* polysaccharides, which can effectively induce the regeneration of hair follicles in healing skin tissue and shorten the wound healing process [180]. The composing films were prepared by *Falkenbergia rufolanosa* polysaccharides and *Trigonella foenum graecum* polysaccharides with PVA in different proportions. The antioxidant activity of the films was evaluated for its effect on wound healing in burned rats [181,182]. Histological examination showed that the composed films with a high proportion of polysaccharides had the highest antioxidant activity. The films could show less erythema, scab, and high collagen content during treatment, which proved that the composed films promoted rapid wound healing and tissue regeneration through their antioxidant activity. *Linum usitatissimum* polysaccharide emulsion and *pimpinella anisum* polysaccharide gel were evaluated for antioxidant activity in vitro and the healing ability of laser burn wounds in a rat model [183,184]. The results showed that these two polysaccharides could prevent living cells from free radical-mediated oxidative damage, which may help to accelerate wound contraction and tissue remodeling. In addition, exopolysaccharides extracted from lactic acid bacteria have a strong antioxidant capacity and can effectively scavenge superoxide anion free radicals and carbon monoxide free radicals [185]. It can improve the function of hASC cells and potentially promote tissue repair and regeneration.

Natural polysaccharides with biocompatibility, biodegradability, and antioxidant activity can provide glycosaminoglycans in the bone cell-matrix, making them an ideal candidate material in bone tissue engineering [186]. The hydroxypropyl modified chitosan (HPCS) has higher antioxidant activity and biocompatibility than chitosan. Genipin-crosslinked and fucoidan-adsorbed nano-HPCS composite scaffolds can improve the activity of 7F2 osteoblast cells, suggesting that the composite scaffolds can be used as a potential biomaterial for bone tissue engineering [187]. In addition, sulfated *Tamarindus indica* polysaccharides and *Bletilla striata* polysaccharides can also repair oxidative stress-induced cartilage tissue damage [188,189]. These studies may provide new approaches to treating OA.

### 6.3. Application of Natural Polysaccharides in Preparation of Antimicrobial Food Additives/Packaging Materials

Food safety is still a significant challenge today. People are increasingly inclined to use natural antioxidants and antibacterial agents in food storage and preservation. The derivative films of some natural fiber materials have excellent gas barriers and mechanical properties. However, as food packaging materials, the antioxidant function is usually needed to ensure the quality and safety of food, including extending the shelf life of foods, especially those with a high-fat content [190]. Packing natural polysaccharides with antioxidant activity into fiber materials is the critical choice to meet the needs of food packaging. *Chickpea hull* polysaccharides (CHPS) were used to form carboxymethyl cellulose (CMC)-based films (CBF) [191]. The hydrophilic polysaccharides can interact well with CMC and adsorb on CBF due to their similar conformational structure to CMC. The test results of antioxidant, antibacterial, and thermal stability were better than those of the control film, suggesting that CHPS–CMC film can be used as a bio-composite material for food packaging. The antioxidant activity of CMC–CHPS films was enhanced with increasing CHPS concentration, which proved that CHPS is responsible for enhanced free radical scavenging and reducing the capacity of the films. The combination of chitosan and gallic acid was also used in food coatings, which mainly play the role of antioxidant and antibacterial, then protect food safety [192]. In addition, natural polysaccharides can also be combined with proteins to prepare food composite films. Adding natural polysaccharides can significantly enhance the antioxidant performance of the films [193]. It is of specific significance to develop natural polysaccharides with antioxidant activity into food packaging materials. Some natural polysaccharides and their applications in drug delivery, tissue engineering, and food packaging are summarized in Table 2.

## 7. Conclusions

In conclusion, the results of scientometric analysis display that the antioxidant activity of natural polysaccharides has become a hotspot topic. Natural polysaccharides and their derivatives are widely used for medicinal and biomedical applications, such as drug delivery and tissue engineering. The antioxidant activity of natural polysaccharides is mainly based on their chemical structure and is affected by the type, source, and extraction method of polysaccharides. Therefore, selecting appropriate chemical structure modification and extraction methods will help to improve the antioxidant activity of polysaccharides. The specific methods need to be further studied, which will help us fully use natural polysaccharides, a rich and safe natural medicinal resource.

## Figures and Tables

**Table 1:** Natural polysaccharides and their antioxidant effects.

Category	Polysaccharides	Main Monosaccharides	Model	Protective Mechanisms	References
Higher plant polysaccharides	*Angelica sinensis* polysaccharides	Glucose, arabinose, and galacturonic acid.	Chondrocyte damage induced by H_2_O_2_.	Protect chondrocyte from H2O2-induced oxidative stress by inhibiting the caspase pathway.	[39]
	*Gastrodia elata* polysaccharides	Glucose	Aging mice induced by D-galactose.	Delay aging by scavenging free radicals.	[66]
	*Lycium barbarum* polysaccharides	Arabinose, galactose, glucose, mannose, and galacturonic acid.	Ultraviolet rays (UVR)-induced HSF cell damage.	Protect HSF cells from UVR damage by regulating Nrf2.	[113]
	*Astragalus* polysaccharides	Mannose, glucose, arabinose, and galactose.	Injury of human umbilical vein endothelial cells induced by H_2_O_2_.	Improve cellular antioxidant capacity and NO bioavailability.	[114]
	*Dendrobium officinale* polysaccharides	Mannose and glucose.	Precancerous lesions of gastric cancer in rats induced by 1-methyl-3-nitro-1-nitrosoguanidine.	Reduce the level of 8-OHdG, and activate the Nrf2 pathway.	[115]
	*Portulaca oleracea* polysaccharides	Rhamnose, arabinose, and galactose.	Neurotoxicity induced by pb.	Protect PC12 cell by reducing the production of ROS and improving cell viability.	[116]
	*Salvia miltiorrhiza* polysaccharides	Glucose, galactose, mannose, and xylose.	Isoproterenol (ISO) -induced myocardial infarction in rats.	Inhibit ISO-induced myocardial injury by enhancing endogenous antioxidant and anti-hyperlipidemia activities.	[117]
	*Momordica charantia* polysaccharides	Rhamnose, galacturonic acid, galactose, xylose, and arabinose.	Streptozotocin (STZ)-induced diabetic rats.	Inhibit oxidative stress and improve the HO-1/Nrf2 pathway to alleviate STZ-induced diabetes.	[118]
	*Pumpkin* polysaccharides	Glycuronate, galactose, and arabinose.	Pancreas β cells in the rat.	Protect pancreatic β cells from oxidative damage by reducing MDA level and increasing SOD activity, then play a hypoglycemic effect.	[119]
Algal polysaccharides	*Enteromorpha prolifera* polysaccharides	Xylose, rhamnose, glycuronate, glucuronic acid, and galactose.	Oxidative damage of Caenorhabditis elegans induced by UV.	Improve the accumulation of ROS and DNA damage in cells by down-regulating miR-48 and miR-51, and up-regulating the expression of SKN-1 and DAF-16.	[16]
	*Ulva pertusa* polysaccharides	Rhamnose, galactose, glucose, and xylose.	Oxidative damage of RAW264.7 cells induced by H_2_O_2_.	Up-regulate the expression of antioxidant enzymes.	[75]
	*Sargassum horneri* polysaccharides	Fucose, galactose, xylose, and glucuronic acid.	Oxidative damage of RAW264.7 cells induced by H_2_O_2_.	Reduce the ROS, NO, and MDA levels in cells and restore the activities of SOD and GSH-px.	[120]
Microbial polysaccharides	*Lentinus edodes* polysaccharides	Xylose, glucose, and galactose.	H22 tumor-bearing mice.	Increase the activities of SOD, CAT, and GSH-px, and enhance the expression of IL-2, TNF-α, and VEGF.	[121]
	*Flammulina velutipes* polysaccharides	Mannose, glucose, galactose, and xylose.	Aging mice induced by D-galactose.	Increase the activity of antioxidant enzymes, reduce lipid peroxidation, and improve inflammation and the state of aging.	[122]
	*Grifola frondose* polysaccharides	Glucose, galactose, and mannose.	Natural aging rats.	Improve memory impairment and histological changes in aged rats by increasing antioxidant enzyme activity.	[123]
	*Ganoderma lucidum* polysaccharides	Glucose, mannose, rhamnose, galactose, and arabinose.	Doxorubicin (DOX)-induced cardiotoxicity.	Inhibit DOX-induced oxidative stress by stabilizing the expression of Nrf2 in H9c2 cells and inhibiting cell apoptosis.	[124]
Animal polysaccharides	*Rana chensinensis* polysaccharides	Glucose, galactose, and mannose.	Aging mice induced by D-galactose.	Improve the activities of antioxidant enzymes in the liver and serum, improve the total antioxidant capacity, and decrease MDA levels.	[110]
	Chitosans	Glucosamine, N-acetylglucosamine.	Human serum albumin is exposed to peroxy free radicals.	Protect albumin from oxidation by scavenging peroxy free radicals.	[112]
	*Apostichopus japonicus* polysaccharides	Glucosamine, galactosamine, glycuronate, mannose, glucose, galactose, and fucose.	Hyperlipidemia rats.	Reduce the triglycerides and low-density lipoprotein, and increase the high-density lipoprotein cholesterol.	[125]

**Table 2:** Natural polysaccharides and their applications.

Applications	Polysaccharides	Composite Materials /Drug Loaded	References
Drug delivery	*Bletilla striata* polysaccharides	Dopamine conjugate with adhesion, berberine	[152]
	Carboxymethyl *Bletilla striata* polysaccharides	PVA, Polydopamine@Metformin, microcapsules	[194]
	*Falkenbergia rufolanosa* polysaccharides, chitosan	Insulin	[195]
	*Moringa* gum polysaccharides	Carbomer, levofloxacin	[196]
	*Portulaca oleracea* polysaccharides	Alendronate, platinum	[197]
	*Araucaria heterophylla* gum polysaccharides	Sodium Tri Meta Phosphate, curcumin	[198]
	*Lycium barbarum* polysaccharides	5-aminosalicylic acid, platinum	[199]
Tissue engineering	*Ulvan* Polysaccharides	Boric acid, glycerol	[177]
	*Enteromorpha prolifera* Polysaccharides	Boric acid, polyacrylamide	[178]
	*Trigonella foenum graecum* polysaccharides	PVA	[181]
	*Falkenbergia rufolanosa* polysaccharides	PVA	[182]
	Chitosan, *Trigonella foenum graecum* seed polysaccharides	Nano-hydroxyapatite	[186]
	Microalgae sulfated polysaccharides	Fluorenylmethoxycarbonyl, diphenylalanine peptide	[200]
	*Acacia* gum polysaccharides	Polyvinylpyrrolidone, carbopol	[201]
	*Moringa oleifera* seed polysaccharides	Silver	[202]
	Sterculia gum, *psyllium* polysaccharides	acrylic acid, N, N’-methylene-bis-acrylamide, moxifloxacin	[203]
	Chitosan, oxidized *Bletilla striata* polysaccharides	Gallic acid	[204]
Food packaging	*Mushroom* polysaccharides	Cellulose nanofiber	[190]
	*Chickpea hull* polysaccharides	CMC	[191]
	Soybean polysaccharides	*Zataria multiflora Boiss* and *Mentha pulegium* essential oils	[205]
	Sulfated *Cardamine hupingshanensis* polysaccharides	Zein	[206]
	Chitosan, κ-carrageenan, locust bean gum	Betacyanin-rich red pitaya flesh extract, PVA	[207]
	Chitosan	β-acids	[208]
	*Momordica charantia* polysaccharides	Phlorotannin	[209]
	*Gracilaria chouae* polysaccharides	CMC and lysozyme	[210]
	*Pleurotus ostreatus* polysaccharides	*Cudrania tricuspidata* leaf extract	[211]
	Alginate, carboxymethyl chitosan, fucoidan	Anthocyanins	[212]

## Data Availability

Not applicable.
